# Manufacturing and Characterization of Toughened Poly(lactic acid) (PLA) Formulations by Ternary Blends with Biopolyesters

**DOI:** 10.3390/polym10010003

**Published:** 2017-12-21

**Authors:** María Jesús García-Campo, Teodomiro Boronat, Luis Quiles-Carrillo, Rafael Balart, Nestor Montanes

**Affiliations:** 1 Materials Science Division, Technological Institute of Materials, Universitat Politècnica de València, 03801 Alcoy, Alicante, Spain; mjgcampo@gmail.com (M.J.G.-C.); luiquic1@epsa.upv.es (L.Q.-C.); nesmonmu@upvnet.upv.es (N.M.); 2 Manufacturing Engineering Division, Technological Institute of Materials, Universitat Politècnica de València, 03801 Alcoy, Alicante, Spain; tboronat@dimm.upv.es

**Keywords:** blending, poly(lactic acid) (PLA), biopolyesters, mechanical properties, thermal properties

## Abstract

Ternary blends with a constant poly(lactic acid) (PLA) content (60 wt %) and varying amounts of poly(3-hydroxybutyrate) (PHB) and poly(ε-caprolactone) (PCL) were manufactured by one step melt blending process followed by injection moulding, with the main aim of improving the low intrinsic toughness of PLA. Mechanical properties were obtained from tensile and Charpy impact tests. The miscibility and morphology of the system was studied by thermal analysis and field emission scanning electron microscopy (FESEM). The obtained results showed a clear phase separation, thus indicating poor miscibility between these three biopolyesters, i.e., PLA, the continuous component with dispersed PHB and PCL domains in the form of different sphere size. Nevertheless, the high fragility of PLA was remarkably reduced, as detected by the Charpy impact test. In accordance with the decrease in brittleness, a remarkable increase in elongation at break is achieved, with increasing PCL load due to its flexibility; in addition, increasing PCL load provides thermal stability at high temperatures. Thus, tailored materials can be manufactured by melt blending PLA, PHB, and PCL in different percentages to offer a wide range of biodegradable polymer blends.

## 1. Introduction

In the last decades, increasing environmental concerns and the evident problematics related to petroleum depletion have given rise to attracting recycling and/or upgrading initiatives supported by new legislation. In the field of polymer science, this phenomenon has been particularly important, as the vast majority of commercial and industrial plastics are petroleum-based materials. This situation has led to the development of new polymers with a marked environmental focus. Biodegradable polymers (or what is more accurate, disintegrable in controlled compost soil) can positively contribute to reducing the total amount of plastics left in landfills, which is a high magnitude environmental problem, particularly in the packaging and food industry, due to the large volume to density ratio of plastic parts and components in these sectors (containers, bottles, trays, cups, etc.).

Poly(lactic acid) (PLA) is one of the most promising biopolymers that can be obtained from renewable feedstocks, such as corn starch, sugar beet, tapioca roots, sugarcane, and so on [1]. It has similar properties to some commodity plastics, and its price is continuously becoming cheaper. These properties lead PLA to a very close-to-market position in comparison to other petroleum-based and biodegradable polymers, such as poly(butylene succinate) (PBS), poly(ε-caprolactone) (PCL), and poly(butylene adipate-*co*-terephthalate) (PBAT) [2,3,4], as well as other biobased and biodegradable polymers such as thermoplastic starch, poly(hydroxyalkanoates) (PHAs), protein-based polymers, and so on [5,6,7,8]. Nevertheless, PLA is a highly brittle polymer with very low toughness. On the other hand, its barrier properties are also poorer than other widely used packaging polymers. For these reasons, researchers are trying to find new formulations for industrial applications. Three different approaches are being investigated to overcome these restrictions, i.e., plasticization, copolymerization, and blending [9].

A wide variety of plasticizers have been proposed for PLA formulations with more or less success. They generally provide a decrease in the glass transition temperature, which gives increased chain mobility; consequently, ductile properties are remarkably improved. Nevertheless, other mechanical properties are compromised, i.e., modulus and tensile strength, among others, and the overall effect of plasticizers on toughness is not high. Typical plasticizers for PLA, i.e., oligomeric lactic acid (OLA) [10], citrate esters [11,12,13,14], glycols, and polyglycols [15,16,17,18], among others, are widely used in plasticized PLA formulations. Vegetable oil-derived plasticizers (maleinized-, acrylated-, hydroxylated-, epoxidized-, among other chemically modified vegetable oils) have been successfully used as PLA plasticizers [19]. Nevertheless, their efficiency in terms of plasticization is lower than that provided by conventional plasticizers, as the glass transition temperature is not remarkably reduced but, in contrast, the overall toughness is considerably increased, as reported by Carbonell-Verdu et al. using epoxidized cottonseed oil to toughen PLA [20]. Ferri et al. showed a remarkable improvement on both mechanical ductile properties, i.e., elongation at break and mechanical resistant properties (modulus and tensile strength), by using epoxidized fatty acid esters and maleinized linseed oil. These balanced properties gave a remarkable increase in the impact-absorbed energy [21,22].

Another approach to improve toughness is copolymerization, but this is not a cost-effective solution [23]. The synthesis of different copolymers at laboratory scale has been reported, but their transfer to industry is still complex. Supthanyakul et al. reported the synthesis of a triblock poly(l-lactide-*b*-butylene succinate-*b*-l-lactide) to provide improved toughness to PLA/PBS blends [24]. Wu et al. reported the potential of a synthesized terpolymer from PHB-PLA-PCL, with a flexible behaviour, good biocompatibility, and interesting potential uses for in vivo medical applications [25]. Carrasco et al. reported a remarkable increase in the toughness and thermal stability of PLA by reactive extrusion with a styrene-acrylic multifunctional oligomer due to a branching effect [26].

A third way to increase toughness is by blending. This is a very cost effective way to reduce the intrinsic brittleness of PLA without compromising other mechanical properties to a great extent. A wide variety of binary blends based on PLA have been proposed in recent years with a high number of polymers such as poly(ε-caprolactone) (PCL) [27], poly(butylene succinate) (PBS) [28], poly(butylene adipate (PBAd) [29], poly(amide) (PA) [30,31], thermoplastic starch (TPS) [32], poly(propylene) (PP) [33], soy protein [34], poly(vinyl chloride) (PVC) [35], poly(propylene carbonate) (PPC) [36], poly(butylene adipate-*co*-terephthalate) (PBAT) [37], poly(butylene succinate-*co*-butylene adipate) (PBSA) [38,39], and so on. As the lack of miscibility of PLA with most polymers is one of the main issues to overcome, several approaches have been proposed. On an industrial scale, reactive extrusion has been proven as a cost effective way to partially compatibilize PLA blends with improved toughness [40,41]. Al-Itry et al. reported the excellent compatibilization effect that a multifunctional epoxy styrene-acrylic oligomer (Joncryl^®^ ADR-4368 by BASF, Ludwigshafen, Germany) can have on PLA/PBAT blends [37]. Abdelwahab et al. reported the excellent compatibilization effect that an epoxy styrene-acrylic multifunctional oligomer could have on PLA/PBAT blends with lignin [42]. Rasal et al. worked on improving the toughness of PLA by reactive blending with poly(acrylic acid) (PAA) with interesting applications in consumer products and biomedical devices [43]. In general, biopolyesters are characterized by hydroxyl terminal groups which can readily react with several functionalities. Also, ternary blends represent an interesting technical solution as the use of different polymers can be used to tailor the desired properties. Luckachan et al. developed binary and ternary blends of PLA with PCL and TPS with remarkable improvements on mechanical and thermal performance [32,44]. In a recent work, several soybean oil-based compatibilizers, i.e., epoxidized soybean oil (ESO), epoxidized-acrylated soybean oil (AESO), and maleinized soybean oil (MSO), have demonstrated the interesting compatibilizing effect of these chemically-modified vegetable oils as environmentally friendly compatibilizers in ternary PLA (60 wt %)/PHB (10 wt %)/PCL (30 wt %) blends [31]. This work aims to develop ternary blends based on PLA as the main component and vary the loading of two additional biopolyesters, namely poly(3-hydroxybutyrate)-PHB and poly(ε-caprolactone)-PCL, to improve toughness of neat PLA without any other compatibilization component to obtain an in depth knowledge of the nature of this ternary blend, and to evaluate the effects of these two biopolyesters on the mechanical performance and thermal stability of the developed materials.

## 2. Materials and Methods

### 2.1. Materials

**Scheme 1:**
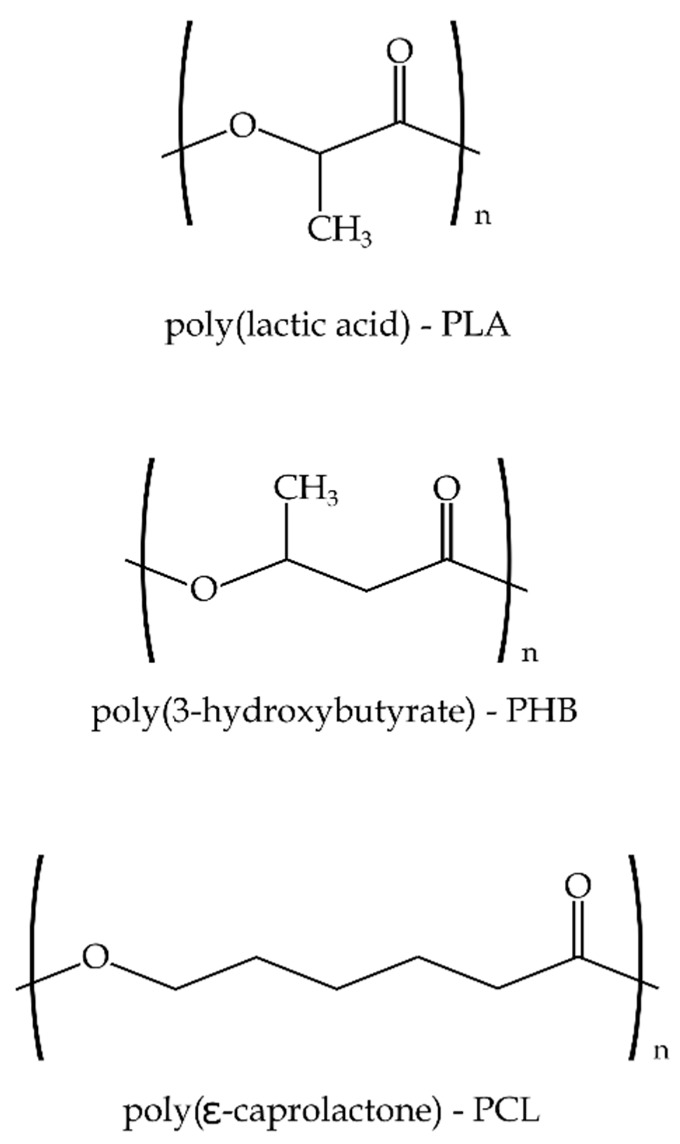
Schematic representation of the chemical structure of poly(lactic acid) (PLA), poly(3-hydroxybutyrate) (PHB), and poly(ε-caprolactone) (PCL).

The PLA used in this work was a commercial grade Ingeo^TM^ Biopolymer 6201D (MFI = 15–30 g/10 min at 210 °C) supplied by NatureWorks (Minnetonka, MN, USA). Its main properties are a density of 1.24 g cm^−3^, a glass transition temperature in the 55–60 °C range, and a melt temperature range comprised between 155–170 °C. Regarding PCL, a commercial grade Capa TM6800 (MFI = 2.01–4.03 g/10 min at 160 °C) was supplied by Perstorp UK Ltd. (Warrington, UK) in pellet form with a density of 1.146 g cm^−3^. PCL main thermal transitions are a glass transition temperature, *T_g_* of −50 to −60 °C, and a melt peak temperature (*T_m_*) in the 58–60 °C range. PHB was a poly(3-hydroxybutyrate) polymer commercial grade P226 (MFI = 10 g/10 min at 180 °C) supplied by Biomer (Krailling, Germany) with a density of 1.25 g cm^−3^. Its main thermal transitions are a *T_g_* of about −5 °C and a melt peak temperature close to 170 °C. The chemical structure of all three biopolyesters is shown in Scheme 1.

### 2.2. Manufacturing of Ternary PLA/PHB/PCL Blends

Manufacturing of blends was carried out in two different stages. Prior to further processing, all materials were dried to avoid moisture, which could affect hydrolysis during manufacturing. PLA and PHB were dried at 60 °C for 24 h, while PCL was dried at 45 °C for 24 h. The amount of PLA in all ternary blends was maintained constant at 60 wt %, as indicated in Table 1, and the results were compared to neat PLA, as the main objective is to obtain toughened PLA formulations. The appropriate amounts of each component were weighed and mechanically pre-mixed in a zipper bag until homogenization. Then, the first manufacturing stage was conducted by extrusion in a twin-screw extruder from Dupra S.L. (Alicante, Spain). The screws had a diameter of 25 mm and a length to diameter (L/D) ratio of 24. A rotating speed of 40 rpm was used with a temperature profile of 175 °C (extrusion die), 170 °C, 165 °C, and 160 °C (feeding hopper). These conditions ensure good processing in terms of viscosity and avoid thermal degradation, as previous studies in the group have revealed. After this mixing stage, the obtained materials were pelletized and further processed by injection moulding in a Mateu & Solé Meteor 75 (Barcelona, Spain) at a temperature profile of 160 °C (hopper), 165 °C, 170 °C, and 175 °C (injection nozzle), using a mirror-finished mold with standard shapes for different tests.

### 2.3. Mechanical Characterization

Mechanical characterization of ternary blends was carried out by standardized tensile and flexural tests in a universal test machine ELIB 50 from Ibertest S.A.E. (Madrid, Spain) equipped with a load cell of 5 kN, following ISO 527-1:2012 and ISO 178:2011, respectively. The crosshead speed rate was adjusted to 5 mm min^−1^, as recommended in the corresponding standards. Surface hardness was measured using the Shore D method in a durometer 676-D from J. Bot Instruments (Barcelona, Spain), following the recommendations of ISO 868:2003. The toughness was estimated by the Charpy impact test with a 1 J pendulum from Metrotec (San Sebastián, Spain), as indicated in ISO 179-1:2010. Impact tests were conducted on notched samples with a “V” type notch and a radius of 0.25 mm. At least 5 different samples were tested for each mechanical test, and the obtained values were averaged. All mechanical tests were carried out at room temperature.

### 2.4. Morphology Characterization

The morphology of the ternary blends was observed by field emission scanning electron microscopy (FESEM) in a ZEIS ULTRA 55 microscope from Oxford Instruments (Abingdon, UK) working at an acceleration voltage of 2 kV. Before placing the samples inside the vacuum chamber for SEM observation, all materials were covered with a thin metallic layer (aurum-palladium) in a sputter coater EMITECH model SC7620, provided by Quorum Technologies, Ltd. (East Sussex, UK). Three different characterizations were made. The first one was on fractured samples coming from impact tests without further preparation. In addition, rectangular samples were subjected to a cryofracture process to observe the morphology without any deformation. Finally, cryofractured surfaces were subjected to a selective extraction process with glacial acetic acid for 1 h, which preferentially attacks PCL and, to a lesser extent, PHB, while PLA remains almost unaltered.

### 2.5. Thermal and Thermomechanical Characterization

The main thermal transitions of the ternary blends were obtained by differential scanning calorimetry (DSC) using a calorimeter from Mettler-Toledo mod. 821 (Schwerzenbach, Switzerland). Samples with an average weight in the 5–7 mg range were placed into standard sealed aluminium crucibles. Two small holes were done on the lids to allow gas scape during the tests. A dynamic program was applied in three different stages: 1st heating stage: from −50 °C up to 200 °C; cooling: 200 °C down to −50 °C; 2nd heating: from −50 °C up to 300 °C. The heating rate was set to 10 °C min^−1^ for all three stages, and the dynamic program was conducted in nitrogen atmosphere (66 mL min^−1^). Thermal degradation was studied by thermogravimetric analysis in a Mettler-Toledo TGA SDTA 851 thermobalance (Schwerzenbach, Switzerland). Samples with a weight in the 5–7 mg range were placed in standard alumina crucibles with a total capacity of 70 µL. A dynamic program was scheduled from 30 °C up to 700 °C at 20 °C min^-1^ in air atmosphere.

Dynamic-mechanical thermal characterization (DMTA) was conducted in an oscillatory rheometer AR-G2 from TA Instruments (New Castle, DE, USA), which was equipped with a special clamp system for solid samples working in a combined shear-torsion mode. The samples, with dimensions of 4 × 10 × 40 mm^3^, were subjected to a temperature sweep from −80 °C up to 120 °C at 2 °C min^−1^, under a maximum deformation (%γ) of 0.1%. The frequency was set to 1 Hz and both the evolution of the storage modulus (G’) and the dynamic damping factor (tan δ) were collected.

## 3. Results and Discussion

### 3.1. Mechanical Properties and Morphology of PLA/PHB/PCL Ternary Blends

**Figure 1:**
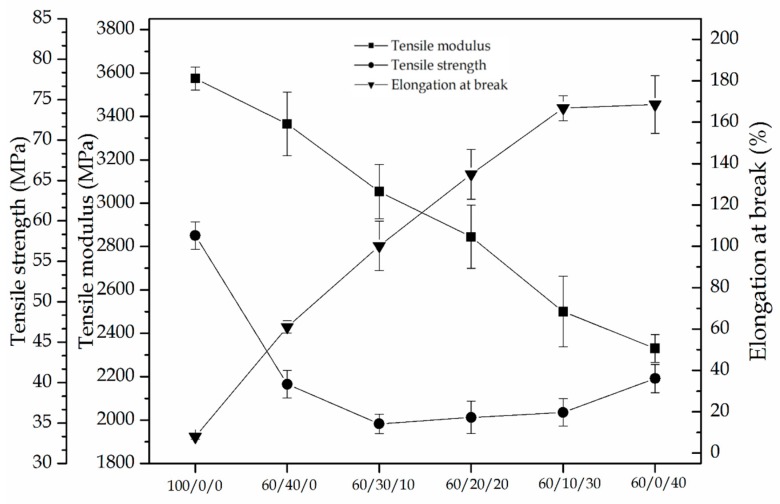
Plot evolution of mechanical tensile properties of PLA/PHB/PCL ternary blends as a function of their composition (wt % PLA/wt % PHB/wt % PCL).

Figure 1 shows the evolution of mechanical tensile properties as a function of the ternary blend composition. Neat PLA is a quite brittle material with a very low elongation at break of 7.87%. This fact, together with a high modulus of about 3.6 GPa and a high tensile strength (58.2 MPa), are responsible for a high intrinsic fragility. Both PHB and PCL have a positive effect on elongation at break, but this effect is more accentuated in the case of PCL, due to its high flexibility compared to PHB. The PLA/PHB/PCL blend (60/40/0) shows a remarkable increase in elongation at break up to 61%, while the modulus is very close to that of neat PLA (around 3.4 GPa), but the tensile strength is noticeably reduced down to 39.8 MPa. Similar trend was observed by Arrieta et al. in PLA/PHB films. In fact, the elongation at break improved by 40% in the PLA/PHB blend containing 25 wt % PHB [45]. Nevertheless, they reported the need for additional plasticization by limonene. With regard to the ternary blend (60/0/40) with 40 wt %, PCL shows a clear effect on ductility with an increase in elongation at break up to 168.5%. These results are in accordance with those reported by Navarro-Baena et al., which achieved an elongation at break of 200% in PLA/PCL blends containing 30 wt % PCL [46]. Chen et al. reported similar findings in PLA/PCL blends, with an increase in elongation at break up to 152.1% for blends containing 40 wt % PCL. The tensile strength is not remarkably reduced and is maintained close to 40 MPa, but the modulus is noticeably reduced down to 2.5 GPa. Intermediate compositions show an intermediate behavior between the binary PLA/PHB and PLA/PCL blends. As one can see, the elongation at break increases with the PCL content, whilst the modulus decreases.

**Figure 2:**
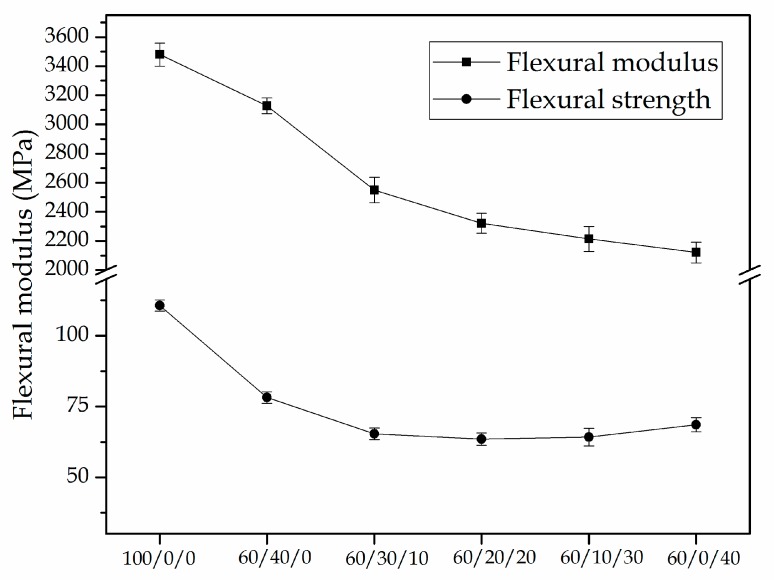
Plot evolution of mechanical flexural properties of PLA/PHB/PCL ternary blends as a function of their composition (wt % PLA/wt % PHB/wt % PCL).

With regard to flexural behavior, Figure 2 shows the evolution of both the flexural modulus and strength. As it can be observed, both flexural properties follow the same tendency, as previously seen in tensile properties. Neat PLA shows a relatively high flexural modulus of about 3.5 GPa, with a flexural strength of 110.7 MPa. The blend that contains only 40 wt % PHB shows similar flexural modulus and an important decrease in flexural strength of about 30% down to values of 78.2 MPa. The blend containing 40 wt % PCL offers the lowest flexural modulus (2.1 GPa), but the flexural strength is slightly higher than intermediate compositions. This could be related to the crystallization process, as both PHB and PCL can potentially affect the overall crystallinity. In fact, PLA crystallization can be highly increased by presence of PCL, as Rizzuto et al. reported, even from PLA in a glassy state [47,48]. The nucleating effect that PCL can provide to formation of PLA crystals has also been proposed, which has a positive effect on increasing mechanical properties [49].

As indicated previously, one of the main drawbacks of PLA is its intrinsic brittleness, which leads to low toughness. In this work, the toughness has been estimated by the Charpy impact test, as summarized in Table 2. The extremely low impact-absorbed values are related to the fact that all samples were previously notched to compare results with the toughened formulations. Neat PLA absorbs very little energy during a sudden impact (1.63 kJ m^−2^). Its ternary blend with 40 wt % PHB (60/40/0) shows a slight increase in toughness, but this can be neglected as it changes up to 1.79 kJ m^−2^. Nevertheless, the ternary blend with 40 wt % PCL (60/0/40) shows a remarkable increase up to values of 6.13 kJ m^−2^, which represents a percentage increase of 276% with regard to neat PLA. Regarding the sole effect of PHB, Zhang et al. reported that the structure of neat PHB is highly affected by the presence of PLA, and this leads to a slight increase in fracture toughness [50]. With regard to the blend with PCL, Chen et al. have reported a remarkable improvement on toughness in PLLA/PDLLA by the addition of PCL in binary blends. They also report an even higher improvement by using a surfactant derived from a copolymer of ethylene and propylene oxide [51].

**Figure 3:**
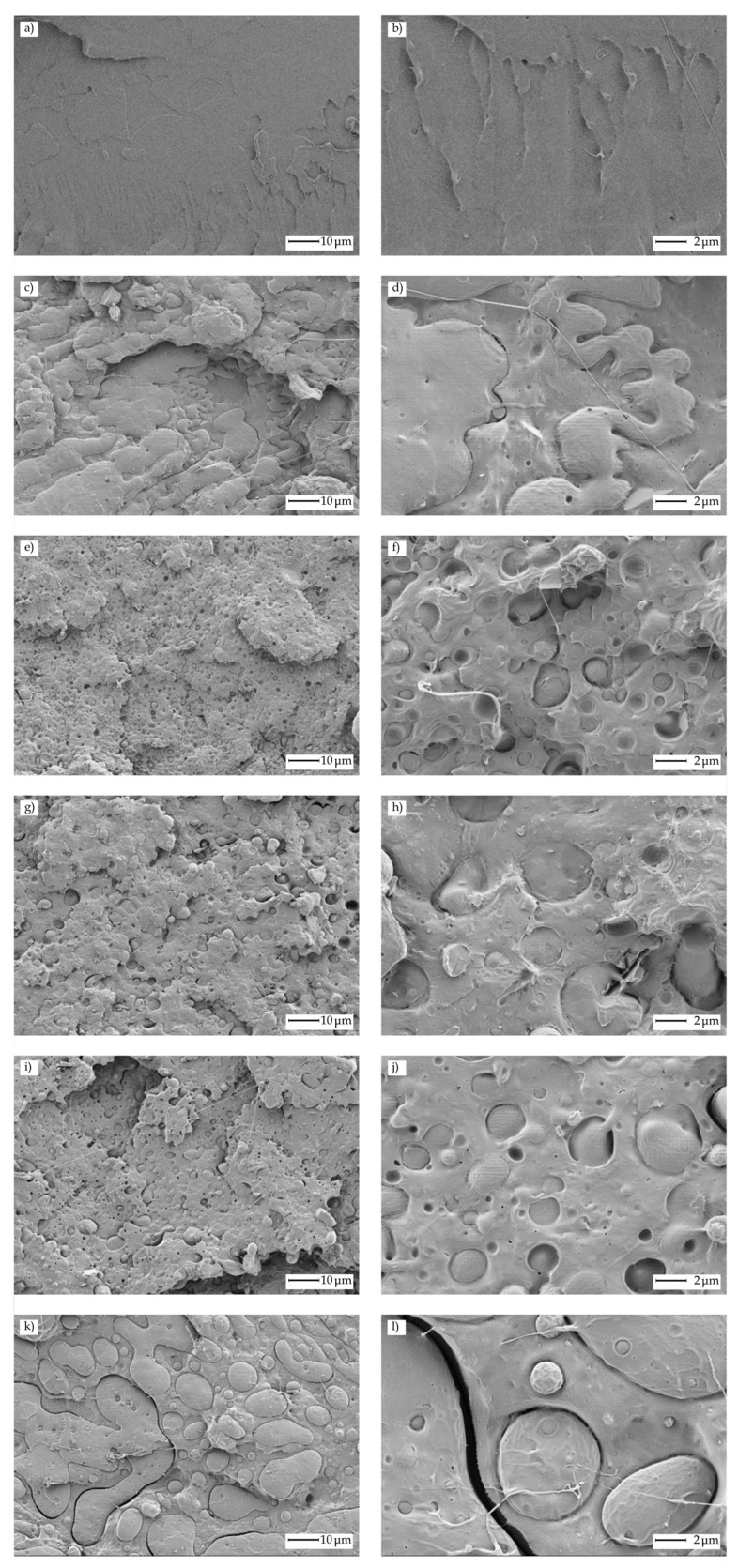
Field emission scanning electron microscopy (FESEM) images of fractured samples from impact test at 1000× (left column) and 5000× (right column) corresponding to PLA/PHB/PCL ternary blends (wt % PLA/wt % PHB/wt % PCL) (**a**,**b**) (100/0/0); (**c**,**d**) (60/40/0); (**e**,**f**) (60/30/10); (**g**,**h**) (60/20/20); (**i**,**j**) (60/10/30); and (**k**,**l**) (60/0/40). The scale bar for left images represents 10 µm while the scale bar for right images stands for 2 µm.

The morphology of the blends shows a clear phase separation with both PHB and PCL, as can be observed in Figure 3. Neat PLA has a brittle fracture with a typical smooth surface with several crack fronts. With regard to the binary PLA/PHB blend (ternary blend 60/40/0), (Figure 3c,d), FESEM image reveals phase separation with a particular morphology. As can be seen, the typical droplet structure is not clearly detected. In contrast, formation of curved shapes attributable to the dispersed PHB domains is observed. This leads to a rougher surface that is remarkably different from that of the neat PLA. This phase separation has been observed in other binary PLA/PHB blends [52,53]. Regarding the blend with PCL (ternary blend 60/0/40), Figure 3k,l show the typical droplet phase separation structure, with a PCL rich phase dispersed in the PLA matrix. PCL-rich droplets show the typical spherical shape, with average diameters ranging from 1 to 5 µm. This droplet structure gives evidence of their immiscibility, as reported by Ferri et al. [27] and Cardona et al. [46], who reported a PCL dispersed phase with a size ranging from 1 to 10 µm in a similar way, as obtained in the herein developed materials. All intermediate compositions show a rough fracture surface with a droplet-like structure in which PCL- and PHB-rich domains are finely dispersed into the PLA matrix. Although phase separation is detectable, the finely dispersed PHB (more fragile) and, particularly, PCL phase (more rubbery) have a positive effect on improved toughness, as indicated previously, as PCL exerts similar effects to a rubber phase in a conventional high impact poly(styrene) [54]. PCL-rich domains seem to be smaller when PHB is present in the ternary blends. Recently, García-Campo et al. have revealed the remarkable improvement in mechanical performance of PLA/PHB/PCL (60 wt %/10 wt %/30 wt %) by using soybean oil-derived compatibilizer agents, thus widening the potential of these ternary blends [31].

**Figure 4:**
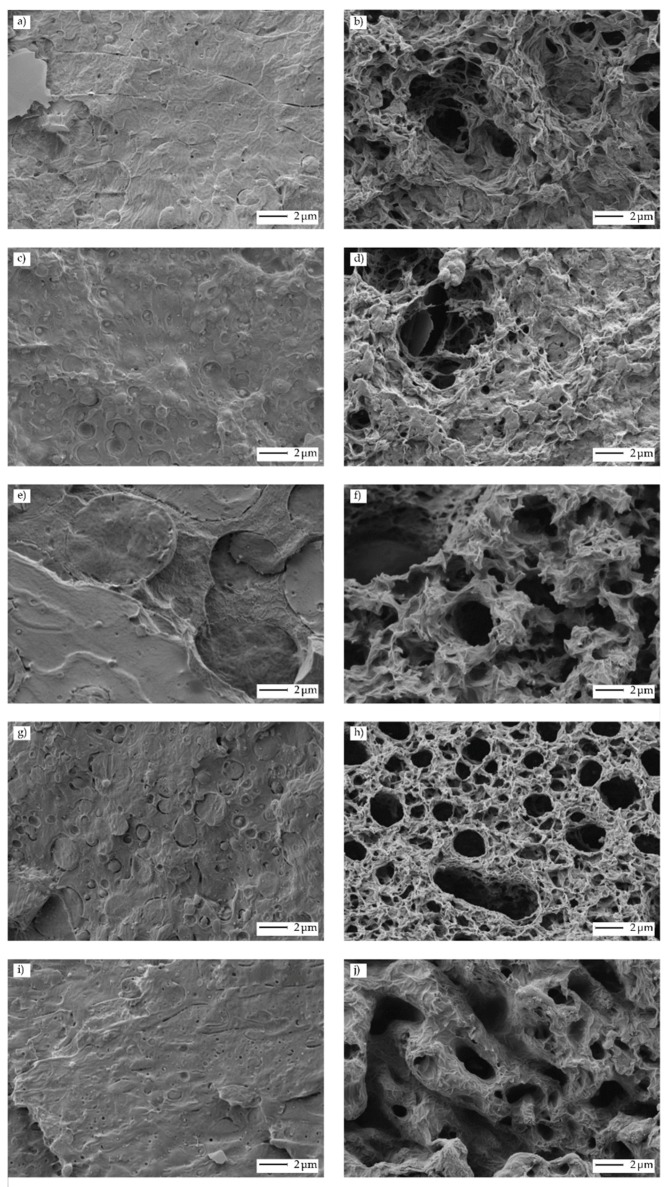
Field emission scanning electron microscopy (FESEM) images of cryofractured samples without chemical attack (**left column**) and with chemical attack (**right column**) at 5000× corresponding to PLA/PHB/PCL ternary blends (wt % PLA/wt % PHB/wt % PCL) (**a**,**b**) (100/0/0); (**c**,**d**) (60/40/0); (**e**,**f**) (60/30/10); (**g**,**h**) (60/20/20); (**i**,**j**) (60/10/30); and (**k**,**l**) (60/0/40). The scale bar represents 2 µm.

Although FESEM images in Figure 3 show a clear phase separation, additional microscopic characterization has been carried out. Figure 4 shows cryofractured surfaces (left images) and cryofractured surfaces subjected to selective extraction with acetic acid, which preferentially dissolves PHB and PCL. The phase separation is detectable on cryofractured surfaces (Figure 4 (left)), but the absence of plastic deformation does not allow to observe the finely dispersed droplets. As can be seen in Figure 4 (right), acetic acid attacks, preferentially, the dispersed phase.

As all compositions contain 60 wt % PLA, both PHB and PCL are removed, thus leading to a rough surface that clearly reveals the phase separation. Nevertheless, this highly rough structure also suggests some miscibility between PLA and PHB. The blend of PLA with 40 wt % PHB (60/40/0) shows an extremely high rough surface, which suggests some miscibility between these two polymers. If total immiscibility between PLA and PHB occurred, the remaining structure after the selective attack would be composed of holes with a smooth surface due to the lack of interactions between them. As can be seen in Figure 4b, the holes are clearly detectable, but their surface is not smooth. In contrast, they offer a very rough surface, which could be related to selective extraction of PHB-rich domains in contact with the PLA main phase. D’Amico et al. reported very low miscibility between PLA and PHB, as slight changes in the *T_g_* values were obtained. They also observed the important role of a conventional and biobased plasticizer, namely tributyrin in partial compatibilization [55]. This situation is different in the blends containing 40 wt % PCL (60/0/40). The cryofractured surface (Figure 4i) reveals clear phase separation, but the chemically attacked surface shows a PLA matrix with less roughness than the blend with PHB (100/40/0) (Figure 4j). This could confirm that PLA and PCL are more immiscible.

### 3.2. Thermal Transitions and Thermo-Mechanical Behaviour of PLA/PHB/PCL Ternary Blends

The main thermal transitions of all three polymers overlap and cannot be clearly identified by conventional (non-modulated) differential scanning calorimetry. Figure S1 in Supplementary Materials shows a comparison of the DSC thermal profiles and the assignment of some thermal transitions.

**Figure 5:**
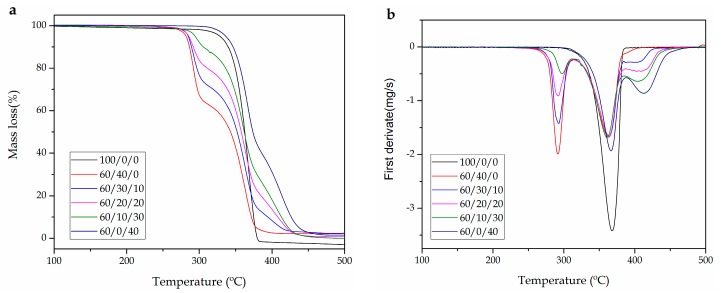
Comparative plot of the thermal degradation of PLA/PHB/PCL ternary blends with different compositions (wt % PLA/wt % PHB/wt % PCL), (**a**) TGA (thermograms), and (**b**) DTG (first derivative curves).

Thermal degradation by thermogravimetric analysis (TGA) also gives interesting results that give support to some previous outcomes. Figure 5 gathers the TGA profiles (Figure 5a) and their corresponding first derivative DTG (Figure 5b) curves, corresponding to the developed PLA/PHB/PCL ternary blends. It is worthy to note the opposite effect that PCL and PHB provide to the ternary blends, in terms of their thermal stability at high temperatures. Neat PLA (100/0/0) degrades in a one-step stage with typical representative temperatures of 328.5 °C (*T*_5_, temperature at which a 5 wt % loss occurs) and 368.5 °C (*T_max_*, which stands for the maximum degradation rate), as summarized in Table 3. The first noticeable fact is that PCL leads to increased thermal stability at high temperatures. The blend containing 40 wt % PCL (60/0/40) shows a two-step degradation process, the first one corresponding to PLA degradation and the second one, at higher temperatures, corresponding to PCL decomposition. As can be seen, the PLA degradation is delayed with the presence of PCL. In fact, the *T*_5_ is moved to 336 °C. Patrício et al. reported the thermal stabilization that PCL provides to PLA. They reported an increase in the characteristic peak degradation temperature of PLA from 325 °C up to 334 °C for PCL/PLA blends, with compositions of 50/50 and 70/30 (*wt*/*wt*), respectively [56]. Mofokeng et al. reported slight changes in the degradation peak temperature of PLA in blends with 30 wt % PCL, but the onset degradation temperature moved towards higher temperatures. They suggested the lack of miscibility between PLA and PCL, as the weight loss in the blends occurred in a two-step process and the amount of mass loss in each stage was directly related to the corresponding polymer content [57]. As can be seen in Table 3, similar results are obtained with regard to neat PLA (100/0/0) and the blend with 40 wt % PCL (60/0/40), as the *T_max_* values for PLA remain almost constant, thus giving evidence of poor miscibility between PLA and PCL. With regard to the blend containing 40 wt % PHB (60/40/0), it is worthy to note that neat PHB is characterized by a remarkably lower thermal stability. As reported by Arrieta et al., its onset degradation temperature (*T*_5_) is close to 167 °C [58]. They also observed a decrease in the onset degradation temperatures for several blends of PHB with PLA. Similar results are obtained with the developed PLA/PHB/PCL ternary blends. As can be seen in Table 3, the *T*_5_ for neat PLA decreases from 328.5 °C down to 281.8 °C for the blend containing 40 wt % PHB (60/40/0), which represents a temperature decrease of almost 50 °C. As the PHB content decreases and the PCL content increases, the overall thermal stability increases. Ternary PLA/PHB/PCL blends degrade in a three-step process, each one corresponding to the individual polymer, and the weight loss for each step is directly related to the corresponding polymer in the blend.

**Figure 6:**
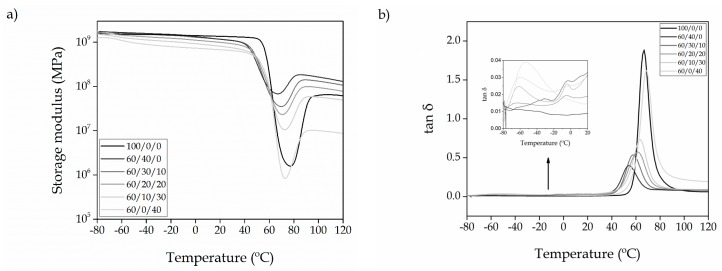
Plot comparison of the dynamic mechanical thermal behavior of PLA/PHB/PCL ternary blends with different compositions (wt % PLA/wt % PHB/wt % PCL); (**a**) storage modulus, G’; and (**b**) damping factor, tan δ.

Dynamic mechanical thermal analysis (DMTA) is a more sensitive technique to evaluate the changes in the *T_g_* temperature range which is, in turn, directly related to miscibility between components in a blend. It also allows estimating some dynamic mechanical properties as a function of temperature. Figure 6a gathers the plots of the evolution of the storage modulus (G’) in terms of temperature for neat PLA and the different ternary PLA/PHB/PCL blends. Three different phenomena can be clearly observed in these plots. The first one is the glass transition temperature of PLA, which is located between the 50–70 °C range. In this, a threefold decrease in the storage modulus occurs. The second important process related to PLA is its cold crystallization, which occurs (for neat PLA) between 80 °C–105 °C, and it is related to the packing process of PLA polymer chains to an ordered structure. The packed structure is more rigid, and thus the storage modulus increases again (by two orders of magnitude). The third process is related to the glass transition temperatures of PHB and PCL that can be observed as a slight decrease in the storage modulus curve at about −50 °C and −5 °C, respectively. Despite this, the glass transition temperatures for PCL- and PHB-rich phases are not clearly resolved by following the plot of the storage modulus. As can be seen in Figure 6a, the presence of PHB or PCL affects, in a remarkable way, the cold crystallization process, as well as the glass transition temperature. Ternary PLA/PHB/PCL blends with PHB show decreased *T_g_* values as the storage modulus curves are moved towards lower temperatures, as reported by Pachekoski et al., which suggests some partial miscibility between PLA and PHB due to the decrease in *T_g_* values through DMTA analysis [59]. With regard to the crystallization, as observed by DSC analysis, PHB addition to PLA leads to crystallization inhibition, as previously reported by Lim et al. in PLA blends with different poly(hydroxyalkanoates) and copolymers [60]. This is in agreement with the results obtained by DMTA. Ternary blends with high PHB content are characterized by a slight crystallization process (an increase in G’ of less than one order of magnitude). With regard to the *T_g_*, Figure 6b shows clear evidence of the effect of each individual polymer (PHB or PCL) on PLA through the dynamic damping behavior. The partial miscibility of PHB with PLA is clearly assessed by a remarkable decrease in *T_g_* from 66 °C for neat PLA (100/0/0) down to 54 °C for the ternary blend with 40 wt % PHB and no PCL (60/40/0). As the PCL content increases up to 10, 20, and 30 wt %, the *T_g_* of PLA is moved towards higher values, as PCL is not as miscible with PLA as with PHB, leading to values of 58 °C (60/30/10), 62 °C (60/20/20), and 64 °C (60/10/30). The blend with 40 wt % PCL and no PHB (60/0/40) shows a slight increase in *T_g_* (69 °C) due to chain mobility restriction. The damping factors in the temperature ranges corresponding to the glass transition of PCL and PHB can be observed in Figure 6b—inlet zoom graph. As can be seen, the *T_g_* range for PCL is located between −60 °C and −50 °C, and the *T_g_* range for PHB is between −15 °C and 0 °C.

## 4. Conclusions

PLA/PHB/PCL ternary blends represent a technical solution to improve the low intrinsic toughness of PLA. PHB and PCL can contribute to different phenomena with an overall positive effect on mechanical and thermal properties of ternary PLA blends. PHB shows partial miscibility with PLA, and this is evidenced by a decrease in the *T_g_* value of PLA from 66 °C down to 54 °C for the ternary blend with 60 wt % PLA/40 wt % PHB/0 wt % PCL. PHB inhibits the cold crystallization process of PLA. On the other hand, PCL contributes positively to improve the thermal stability of the ternary blends. As observed by thermal analysis, some partial miscibility between PLA and PHB is evident, whilst very poor miscibility between PLA and PCL is detected. Nevertheless, the finely dispersed PCL-rich phase positively contributes to increased toughness up to values of 6.13 kJ m^−2^ for the ternary blend with 60 wt % PLA/0 wt % PHB/40 wt % PCL, which is a remarkably superior value to that of neat PLA (1.63 kJ m^−2^). Although PCL provides the highest toughness values, other mechanical properties are remarkably reduced; for this reason, PHB can compensate for this decrease, thus leading to balanced mechanical and thermal properties. This work represents an environmentally friendly solution to overcoming the low intrinsic toughness of PLA. By combining the appropriate amounts of each biopolyester, it is possible to obtain tailored properties.

## Figures and Tables

**Table 1:** Composition and labelling of PLA/PHB/PCL ternary blends.

Code	PLA (wt %)	PHB (wt %)	PCL (wt %)
100/0/0	100	0	0
60/40/0	60	40	0
60/30/10	60	30	10
60/20/20	60	20	20
60/10/30	60	10	30
60/0/40	60	0	40

**Table 2:** Mechanical properties (Charpy impact test on notched samples and Shore D hardness) of PLA/PHB/PCL ternary blends as a function of their composition (wt % PLA/wt % PHB/wt % PCL).

PLA/PHB/PCL (wt %)	Impact-Absorbed Energy (kJ m^−2^)	Shore D Hardness
100/0/0	1.63 ± 0.14	69.0 ± 1.2
60/40/0	1.79 ± 0.10	69.6 ± 0.9
60/30/10	2.40 ± 0.38	69.3 ± 0.7
60/20/20	2.45 ± 0.62	67.0 ± 0.9
60/10/30	5.06 ± 0.94	65.0 ± 1.0
60/0/40	6.13 ± 0.21	65.2 ± 0.3

**Table 3:** Thermal degradation properties of PLA/PHB/PCL blends with different compositions (wt % PLA/wt % PHB/wt % PCL) obtained by TGA characterization.

PLA/PHB/PCL (wt %)	*T*_5_ (°C)	*T_max_* (°C)			Residual Weight (%)
		PHB	PLA	PCL	
100/0/0	328.5 ± 2.6	-	368.5 ± 0.9	-	0.36 ± 0.12
60/40/0	281.8 ± 2.9	291.5 ± 1.2	363.2 ± 0.7	390.6 ± 1.2	4.49 ± 0.24
60/30/10	284.8 ± 3.3	292.5 ± 0.9	363.8 ± 0.8	403.2 ± 1.6	4.23 ± 0.23
60/20/20	285.5 ± 3.4	291.8 ± 1.1	363.6 ± 0.8	408.1 ± 1.3	2.73 ± 0.41
60/10/30	294.3 ± 3.1	297.4 ± 0.6	360.6 ± 1.1	407.4 ± 0.9	1.82 ± 0.54
60/0/40	336.0 ± 2.4	-	366.3 ± 0.6	413.3 ± 0.7	2.54 ± 0.35

## Supplementary Materials

The following are available online at www.mdpi.com/2073-4360/10/1/3/s1, Figure S1: Comparative DSC thermograms of PLA/PHB/PCL ternary blends with different compositions (wt % PLA/wt % PHB/wt % PCL).
