# Supplementary material for: Manufacturing and Characterization of Toughened Poly(lactic acid) (PLA) Formulations by Ternary Blends with Biopolyesters
Source: Polymers (Basel). 2017 Dec 21;10(1):3. doi: 10.3390/polym10010003 (PMC6415117; doi:10.3390/polym10010003)
Supplement: Supplementary file 1 [file polymers-10-00003-s001.pdf]

polymers

MDPI

Supplementary Information

Manufacturing and Characterization of Toughened Poly(lactic acid) (PLA) Formulations by Ternary Blends with Biopolyesters

María Jesús García-Campo [1], Teodomiro Boronat [2], Luis Quiles-Carrillo [1], Rafael Balart [1,*] and Nestor Montanes [1]

[1] Materials Science Division, Technological Institute of Materials, Universitat Politècnica de València, 03801 Alcoy, Alicante, Spain; mjgcampo@gmail.com (M.J.G.-C.); luiquic1@epsa.upv.es (L.Q.-C.); nesmonmu@upvnet.upv.es (N.M.)

[2] Manufacturing Engineering Division, Technological Institute of Materials, Universitat Politècnica de València, 03801 Alcoy, Alicante, Spain; tboronat@dimm.upv.es

* Correspondence: rbalart@mcm.upv.es; Tel.: +34-966-52-8400

A1: Characterization of thermal transitions by Differential Scanning Calorimetry

Figure S1 gathers the DSC thermograms of neat PLA and its ternary blends with PHB and PCL. Neat PLA (100/0/0) shows all three main transitions. The glass transition temperature, T_g, is located at about 65.0 ºC. The melt temperature peak (T_m) is close to 171.4 ºC and the cold crystallization is evident as an exothermic broad peak comprised between 90 – 120 ºC with a peak (T_{cc}) located at 108.8 ºC. The blend with 40 wt.% PHB (60/40/0), shows some interesting changes in the thermal transitions. In particular, it is worthy to note that the cold crystallization of PLA, disappears. This indicates that PHB chains restrict chain mobility and thus, PLA chains cannot rearrange in a packed-ordered way. Depending on the molecular weight, the effect on the cold crystallization of PLA is more or less pronounced.

The melt temperature is not highly affected but the melt peak in Figure S1 for the blend (60/40/0), shows two overlapping melting processes, corresponding to PLA and PHB. With regard to the T_g of PLA, it decreases down to 55 ºC but it is not clearly detectable by conventional DSC. The use of modulated DSC could be helpful to separate both the PCL melt peak (which involves a melt enthalpy) and the PLA glass transition temperature related to a change in its heat capacity. Conventional DSC is useful to evaluate the processing window as well as the upper service temperature. More detailed information about thermal transitions will be given by using dynamic mechanical-thermal analysis (DMTA). The T_g of PHB is located below 0 ºC but it cannot be clearly detected by conventional DSC analysis. With regard to the blend with 40 wt.% PCL (60/0/40), the DSC thermograms shows some differences. The melt peak of PCL can be observed at about 60 ºC and overlaps with the T_g of PLA. Then, the cold crystallization process is detectable as a small broad peak (due to the dilution effect in the blend) at the same position as in neat PLA. This indicates that PCL does not affect the crystal structure of PLA which could suggest high immiscibility between them.

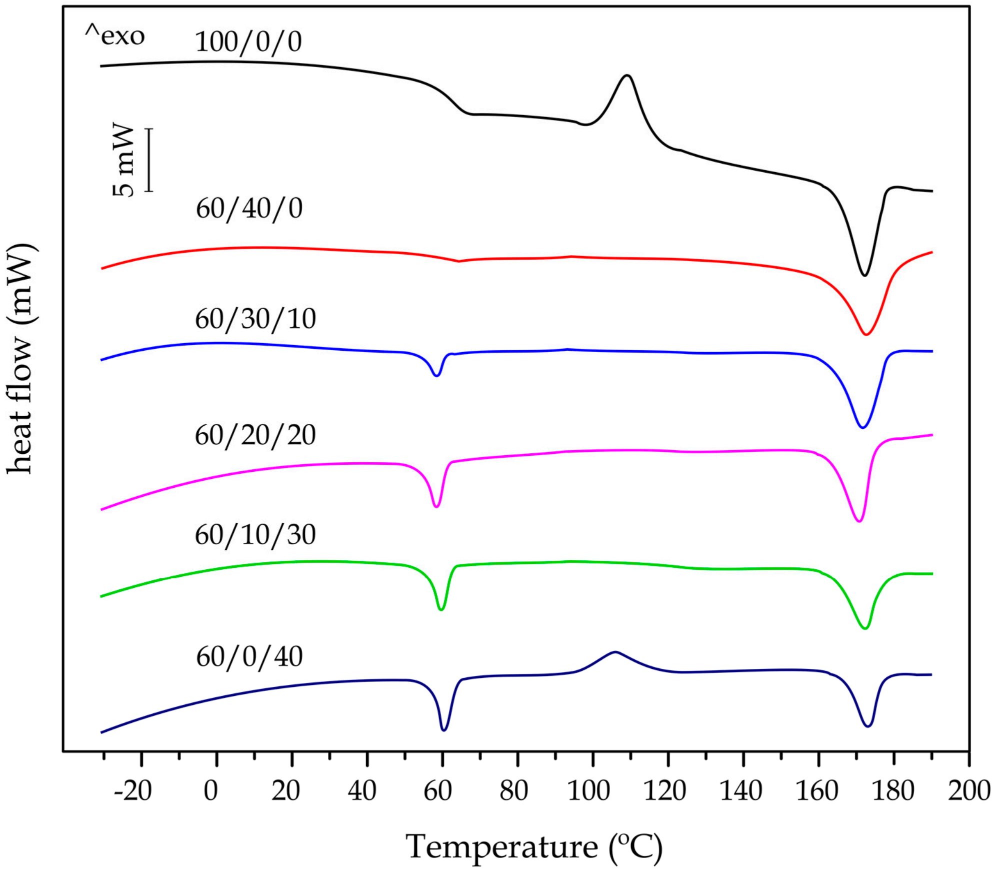

Figure S1. Comparative DSC thermograms of PLA/PHB/PCL ternary blends with different compositions (wt.% PLA/ wt.% PHB/ wt.% PCL**).**
